# Latent Class Analysis Identifies Distinct Phenotypes of Systemic Lupus Erythematosus Predictive of Flares after mRNA COVID-19 Vaccination: Results from the Coronavirus National Vaccine Registry for ImmuNe Diseases SINGapore (CONVIN-SING)

**DOI:** 10.3390/vaccines12010029

**Published:** 2023-12-27

**Authors:** Tao Ming Sim, Manjari Lahiri, Margaret Ma, Peter Pak-Moon Cheung, Anselm Mak, Warren Fong, Stanley Angkodjojo, Chuanhui Xu, Kok Ooi Kong, Thaschawee Arkachaisri, Kee Fong Phang, Teck Choon Tan, Qai Ven Yap, Yiong Huak Chan, Melonie Sriranganathan, Tyng Yu Chuah, Nur Emillia Roslan, Yih Jia Poh, Annie Law, Amelia Santosa, Sen Hee Tay

**Affiliations:** 1 Yong Loo Lin School of Medicine, National University of Singapore, Singapore 117599, Singapore; e0268883@u.nus.edu (T.M.S.); 2 Division of Rheumatology, Department of Medicine, National University Hospital, 1E Kent Ridge Road, Level 10, NUHS Tower Block, Singapore 119228, Singapore; margaret_ma@nuhs.edu.sg (M.M.); amelia_santosa@nuhs.edu.sg (A.S.); 3 Department of Medicine, Yong Loo Lin School of Medicine, National University of Singapore, Singapore 117597, Singapore; warren.fong.w.s@singhealth.com.sg (W.F.); kee_fong_phang@nuhs.edu.sg (K.F.P.); 4 Department of Rheumatology and Immunology, Singapore General Hospital, Singapore 169608, Singapore; 5 Duke-NUS Medical School, Singapore 169857, Singapore; thaschawee.arkachaisri@singhealth.com.sg; 6 Rheumatology Service, Department of General Medicine, Sengkang General Hospital, Singapore 544886, Singapore; 7 Department of Rheumatology, Allergy and Immunology, Tan Tock Seng Hospital, Singapore 308433, Singapore; chuanhui_xu@ttsh.com.sg (C.X.);; 8 Rheumatology and Immunology Service, Department of Paediatric Subspecialties, KK Women’s and Children’s Hospital, Singapore 229899, Singapore; 9 Chronic Programme, Alexandra Hospital, Singapore 159964, Singapore; 10 Division of Rheumatology, Department of General Medicine, Khoo Teck Puat Hospital, Singapore 768828, Singapore; 11 Biostatistics Unit, Yong Loo Lin School of Medicine, National University of Singapore, Singapore 117599, Singapore; qaiven@nus.edu.sg (Q.V.Y.); medcyh@nus.edu.sg (Y.H.C.); 12 Division of Rheumatology, Department of Medicine, Changi General Hospital, Singapore 529889, Singapore

**Keywords:** COVID-19, vaccination, flare, systemic lupus erythematosus

## Abstract

We recently reported that messenger ribonucleic acid (mRNA) coronavirus disease 2019 (COVID-19) vaccination was associated with flares in 9% of patients with systemic lupus erythematosus (SLE). Herein, we focused our analysis on patients from a multi-ethnic Southeast Asian lupus cohort with the intention of identifying distinct phenotypes associated with increased flares after mRNA COVID-19 vaccination. Methods: Six hundred and thirty-three SLE patients from eight public healthcare institutions were divided into test and validation cohorts based on healthcare clusters. Latent class analysis was performed based on age, ethnicity, gender, vaccine type, past COVID-19 infection, interruption of immunomodulatory/immunosuppressive treatment for vaccination, disease activity and background immunomodulatory/immunosuppressive treatment as input variables. Data from both cohorts were then combined for mixed effect Cox regression to determine which phenotypic cluster had a higher risk for time to first SLE flare, adjusted for the number of vaccine doses. Results: Two clusters were identified in the test (C1 vs. C2), validation (C1′ vs. C2′) and combined (C1″ vs. C2″) cohorts, with corresponding clusters sharing similar characteristics. Of 633 SLE patients, 88.6% were female and there was multi-ethnic representation with 74.9% Chinese, 14.2% Malay and 4.6% Indian. The second cluster (C2, C2′ and C2″) was smaller compared to the first. SLE patients in the second cluster (C2 and C2′) were more likely to be male, non-Chinese and younger, with higher baseline disease activity. The second cluster (C2″) had more incident flares (hazard ratio = 1.4, 95% confidence interval 1.1–1.9, *p* = 0.014) after vaccination. A higher proportion of patients in C2″ had immunomodulatory/immunosuppressive treatment interruption for vaccination as compared to patients in C1″ (6.6% vs. 0.2%) (*p* < 0.001). Conclusion: We identified two distinct phenotypic clusters of SLE with different patterns of flares following mRNA COVID-19 vaccination. Caution has to be exercised in monitoring for post-vaccination flares in patients with risk factors for flares such as non-Chinese ethnicity, young age, male gender and suboptimal disease control at the time of vaccination.

## 1. Introduction

The world is navigating and transiting towards co-existing with the severe acute respiratory syndrome coronavirus 2 (SARS-CoV-2) virus. Systemic lupus erythematosus (SLE) is a debilitating autoimmune disease characterised by a myriad of heterogenous and complex clinical manifestations, usually requiring immunosuppressive therapy to achieve remission [1,2]. People with SLE and other autoimmune inflammatory rheumatic diseases have been identified as a vulnerable patient population, with reports of being significantly more susceptible to contracting coronavirus disease 2019 (COVID-19) as compared to the general population and at heightened risk of poorer outcomes. This is likely due to aberrancy in mounting an appropriate immune response to SARS-CoV-2, coupled with underlying immunosuppression from systemic medication [3,4]. In patients with SLE, this is often compounded by a high comorbidity burden due to multi-organ damage including cardiovascular and kidney disease, further exacerbating susceptibility to COVID-19 [5,6]. The Food and Drug Administration approval of messenger ribonucleic acid (mRNA) vaccines from Pfizer-BioNTech^®^ and Moderna^®^ which make use of a novel lipid nanoparticle carrier system led to widespread adoption of mass vaccination programs, particularly in developed countries [7]. 

Universal uptake of vaccination is the cornerstone for effective control of COVID-19 at the population level, which is especially important in safeguarding vulnerable patients. However, concerns regarding suboptimal efficacy during immunosuppressive treatment, the fear of side effects or triggering disease flares after COVID-19 immunisation have been identified as major barriers contributing to vaccine hesitancy amongst SLE patients [8,9]. Indeed, the theoretical risk of inducing disease flares exists, conjectured to be due to production of neutralising antibodies, molecular mimicry or inflammatory response to lipid nanoparticles which bear self-adjuvant properties [10]. In addition, the role of type I interferons (IFN) in the immunopathogenesis of SLE has been well recognised as a core mediator of disease [11]. Toll-like receptor 7 (TLR7) is central in driving the production of type I IFN and promoting activation of autoreactive B cells which underpins SLE pathogenesis [12,13]. Single-stranded mRNA molecules are potential ligands of TLR7, a crucial pattern recognition receptor of the innate immune system which functions to detect single-stranded RNA [14,15]. On a background of overexpressed TLR7 in susceptible SLE patients, it is conceivable that overzealous production of type I IFN in response to mRNA in COVID-19 vaccination can drive disease activity and induce flares. 

Clinical clustering in heterogenous diseases such as SLE has been demonstrated to identify disease subsets and might have value in predicting patterns of disease flares [16]. We aim to identify patient demographics and clinical features that predispose to SLE flares after vaccination, using an unbiased, multidimensional, clustering approach. 

## 2. Materials and Methods

### 2.1. Study Population

The Coronavirus National Vaccine registry for ImmuNe diseases SINGapore (CONVIN-SING) was conducted as a multicentre observational study to determine and evaluate risk factors of disease flares after mRNA COVID-19 vaccination. Details of this retrospective cohort study and electronic health record review have been reported recently [17]. Eight of nine public hospitals in Singapore took part in this observational study. Children 12 years of age or older and adults who had received at least dose of the mRNA COVID-19 vaccine and who had a diagnosis of SLE prior to vaccination were included in this study. There was no exclusion criterion. A list of patients with SLE and other autoimmune rheumatic diseases was extracted using diagnosis codes and matched against the National Immunisation Register for dates of vaccination and type of vaccine doses. Inclusion of patients occurred consecutively, in order of the date of the 1st vaccine dose.

Patients entered the cohort from the date of 1st mRNA vaccine dose and were censored at the time of flare or on the date of the clinic visit which was at least 90 days from cohort entry, whichever came first. Using manual chart review, data were abstracted onto a secure web-based portal and included patient demographics, confirmation of diagnosis, physician-defined baseline disease activity (see below), baseline immunosuppressive treatment, treatment interruption prior to vaccination, severity and date of flare if any, organ system of flare for SLE, hospitalisation for flare and previous diagnosis of COVID-19. Disease activity was assessed using the Physician Global Assessment (PGA), if reported. The PGA is a measure of disease activity on a visual analogue scale with equal markings between 0–3, with higher scores reflecting worse disease activity [18]. Prior to the start of the study, a training session was conducted to standardise data entry across sites. Remission, mild, moderate and high disease activity were defined as PGA scores of 0, 0.1–0.5, 0.6–1.5 and >1.5, respectively. Synchronisation of definitions of flare and pre-vaccine disease activity was undertaken using practice cases.

If there was a flare, additional items were abstracted, such as organs/systems involved, flare severity and whether hospitalisation was required. The severity of SLE flare was determined as follows. A mild, self-limiting flare was defined as one that necessitated no treatment escalation or early consult with the specialist, and resolved before the index visit, though the patient may have taken non-steroidal anti-inflammatory drugs (NSAIDs) or analgesics or self-increased glucocorticoids (GC) for a few days. A mild to moderate, non-self-limiting flare was one that required an earlier specialist consult and/or increase in immunosuppressive treatment, but not exceeding prednisolone 20 mg per day and not requiring intraarticular (IA) or intramuscular (IM) GC injection or new biologic or cytotoxic drug (e.g., cyclophosphamide) initiation. A severe disease flare was one that required hospitalisation or GC more than prednisolone equivalent of 20 mg per day or IM/IA GC, pulsed intravenous GC or new initiation of a cytotoxic agent or biologic. The National Healthcare Group Domain Specific Review Board E (reference code: 2021/0043) provided ethics approval before the commencement of the study. The requirement for patient consent was waived as the data were obtained by review of electronic health record only, without any direct patient contact.

### 2.2. Statistical Analyses

We included all flares within 90 days of the 1st dose of an mRNA vaccine. The public healthcare system in Singapore is divided into three clusters based on the catchment areas served by the hospitals in each cluster. We grouped the 8 hospitals into 2 groups based on healthcare clusters, to serve as test and validation cohorts, respectively, for latent class analysis (LCA). LCA is a statistical method for determining homogeneous but unobservable subgroups (or latent classes) within a population based on patterns of observed variables [19]. LCA places subjects into clusters based on the input variables [20]. 

Baseline characteristics of patients were selected as input variables for the LCA, namely (i) age; (ii) ethnicity; (iii) gender; (iv) vaccine type; (v) past COVID-19 infection; (vi) interruption of immunomodulatory/immunosuppressive treatment for vaccination; (vii) disease activity and (viii) background immunomodulatory/immunosuppressive treatment. The best fitting model was chosen using the Bayesian information criterion (BIC) for each cohort. Cluster stability was examined using discriminant analysis by the leave-out-one method. For continuous variables, we used median (interquartile range, IQR) and for categorical variables we used percentages and frequencies. Differences in continuous variables were assessed using a Mann–Whitney U test, while Fisher’s exact or Chi-square were used for categorical variables. We subsequently merged the test and validation dataset into a combined cohort to assess the association between the clusters identified from LCA and time to first flare using a mixed effect Cox regression model to account for clustering effect of hospital site and further adjusting for flare after 1st or 2nd dose of mRNA COVID-19 vaccine. Kaplan–Meier curves with log-rank test statistics were generated to compare flare-free survival after first dose. Statistical analysis was performed using Stata v18 (StataCorp LLC, College Station, TX, USA) with statistical significance set at 2-sided *p* < 0.05. 

## 3. Results

### 3.1. Demographics and Clincal Characteristics of All SLE Patients

**Figure 1:**
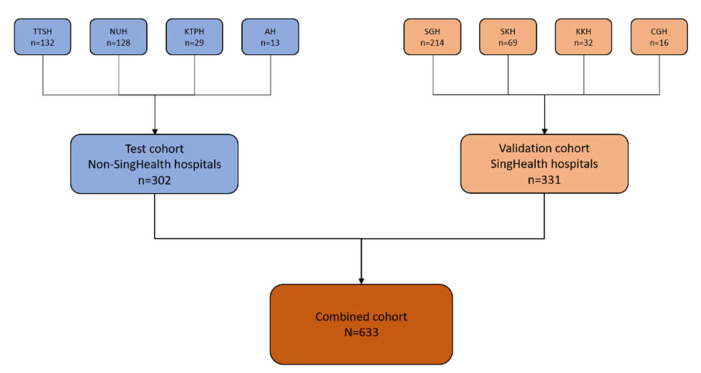
Flow diagram showing the repartitioning of patients from the various hospitals into test and validation cohorts. A total of 633 SLE patients from 8 different hospitals in Singapore were included in the analysis. A total of 302 patients (from non-SingHealth hospitals, colour coded in blue) formed the test cohort and 331 patients (from SingHealth hospitals, colour coded in orange) formed the validation cohort. Abbreviations: National University Hospital, NUH; Tan Tock Seng Hospital, TTSH; Alexandra Hospital, AH; Khoo Teck Puat Hospital, KTPH; Singapore General Hospital, SGH; KK Women’s and Children’s Hospital, KKH; Sengkang Hospital, SKH; Changi General Hospital, CGH.

A total of 633 SLE patients were included from January 2021 to February 2022 in the analysis (Figure 1), with baseline characteristics as depicted in Table 1. The majority of patients were female (88.6%) and there was multi-ethnic representation with 74.9% Chinese, 14.2% Malay and 4.6% Indian. The median age of patients was 52 years (IQR 38–64). 52.0% of the subjects were in remission while 41.7%, 5.7% and 0.6% had low, moderate and high baseline disease activity, respectively. The majority of patients (575/633, 90.8%) received BNT162b2 (Pfizer/BioNTech) mRNA COVID-19 vaccine. 519 (82%) SLE patients were on hydroxychloroquine and 51 (8.1%) were on prednisolone > 7.5 mg/day. Mycophenolate mofetil (24.5%), azathioprine (13.4%) and methotrexate (5.5%) were the most frequent immunosuppressive treatments. Only 2 (0.3%) received rituximab. Treatment was interrupted in 14 (2.2%) patients for vaccination. 10 (1.6%) patients had previous COVID-19 infection. At baseline, the test and validation cohorts were different in terms of age, vaccine type, interruption of immunomodulatory/immunosuppressive treatment for vaccination, disease activity and medication use (e.g., prednisolone, hydroxychloroquine, mycophenolate mofetil, azathioprine and cyclosporine) (Table 1).

### 3.2. Test Cohort

**Figure 2:**
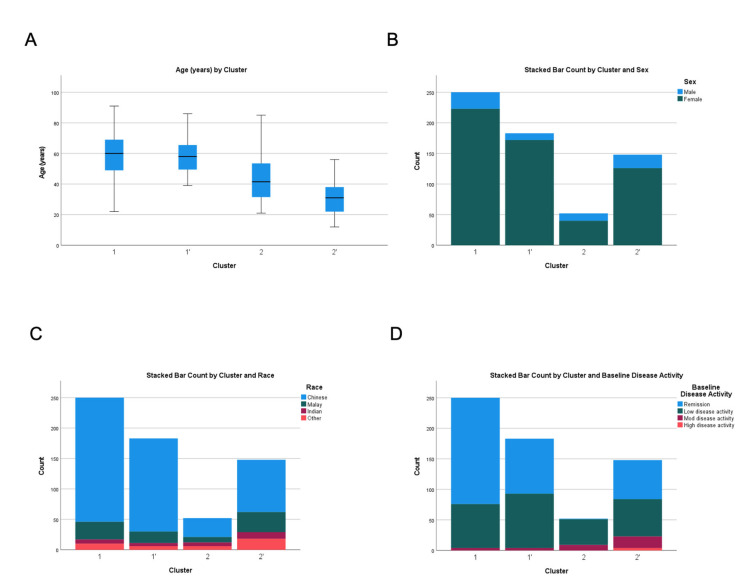
Comparison of (**A**) age, (**B**) gender, (**C**) ethnicity and (**D**) baseline disease activity across test and validation cohorts. C1 and C1′ denote the first cluster of test and validation cohorts, respectively. C2 and C2′ denote the second cluster of the test and validation cohorts, respectively.

Three hundred and two patients from two healthcare clusters (National Healthcare Group and National University Health System) formed the test cohort and after comparing various iterations, a two-cluster model provided the best fit (Table 1 and Table 2 and Supplementary Table S1). Discriminant analysis using the leave-one-out analysis showed correct classification of 96% patients in the test cohort. The median follow-up duration from the first vaccine dose to index clinic visit was 4.4 months (IQR 3.4–5.5). Cluster 1 (C1) was the larger cluster (n = 250, 82.8%). Patients were predominantly female (89.2%), Chinese (81.6%), with median age of 60 years (IQR 49–69) (Figure 2). Cluster 2 (C2) was the smaller cluster (n = 52, 17.2%) with more male patients (23.1%) (*p* = 0.023), younger with a median age of 41.5 (IQR 31.5–53.5) (*p* < 0.001) and more ethnically diverse than C1 (Chinese 59.6%, Malays 17.3%, Indians 11.5% and others 11.5%) (*p* < 0.001) (Figure 2). 

Overall, patients in C1 had better SLE control in terms of baseline disease activity and medication requirement compared to C2. C1 and C2 were significantly different in terms of baseline disease activity: remission (69.6% vs. 1.9%), low disease activity (28.8% vs. 80.8%) and moderate disease activity (1.6% vs. 17.3%) (*p* < 0.001) (Figure 2). Accordingly, SLE patients in Cluster 1 were less likely to be on prednisolone > 7.5 mg/day (1.6% vs. 25.0%) (*p* < 0.001), hydroxychloroquine (74.0% vs. 98.1%) (*p* < 0.001) and immunosuppressants such as cyclosporine (0.4% vs. 19.2%) (*p* < 0.001), methotrexate (3.6% vs. 19.2%) (*p* < 0.001) and tacrolimus (0.0% vs. 11.5%) (*p* < 0.001). 

Information regarding flares is shown in Table 3. SLE patients in C2 were more likely to flare after COVID-19 mRNA vaccination (17.3% vs. 7.6%) (*p* = 0.036) with an incident flare rate of 12.4 per 1000 and 5.4 per 1000 person-months, respectively (*p* = 0.036). Among those who flared, there was no significant difference in time to flare in C1 and C2 (*p* = 0.449). C1 and C2 were significantly different in the severity of SLE flares after vaccination: mild/self-limiting (2.8% vs. 1.9%), mild to moderate (4.0% vs. 13.5%) and severe (0.8% vs. 1.9%) flares (*p* = 0.034). C2 had more haematological flares compared to C1 (5.8% vs. 0.8%) (*p* = 0.037). There was no significant difference in the proportion of SLE patients who flared after the first versus second vaccine doses (1.7% vs. 1.4%, *p* = 0.756) (Supplementary Table S2).

### 3.3. Validation Cohort

Three hundred and thirty-one patients from the Singapore Health Services (SingHealth) hospitals formed the validation cohort and a two-cluster model provided the best fit (Table 1 and Table 2 and Supplementary Table S1). Discriminant analysis using the leave-one-out analysis showed that 96.1% of patients in the validation cohort were correctly classified. The median follow-up duration from the first vaccine dose to index clinic visit was 4.0 months (IQR 3.2–5.2). Clusters 1′ (C1′) and 2′ (C2′) were very similar to C1 and C2 of the test cohort. C2′ was the smaller cluster, with younger, larger proportion of male patients and more ethnically diverse than C1′ (Figure 2). Similarly, patients in C1′ had a better disease control in terms of baseline disease activity and medication requirement compared to C2′ (Table 2). SLE patients in C2′ also tended to flare after COVID-19 mRNA vaccination in terms of prevalence (16.2% vs. 9.3%) (*p* = 0.066) and incidence rate per 1000 person-months (11.6 vs. 6.6) (*p* = 0.066) (Table 3). However, there were no statistical differences in the flares of the different systems assessed. 

### 3.4. Cox Regression Analysis of SLE Flare-Free Survival in the Combined Cohort

**Figure 3:**
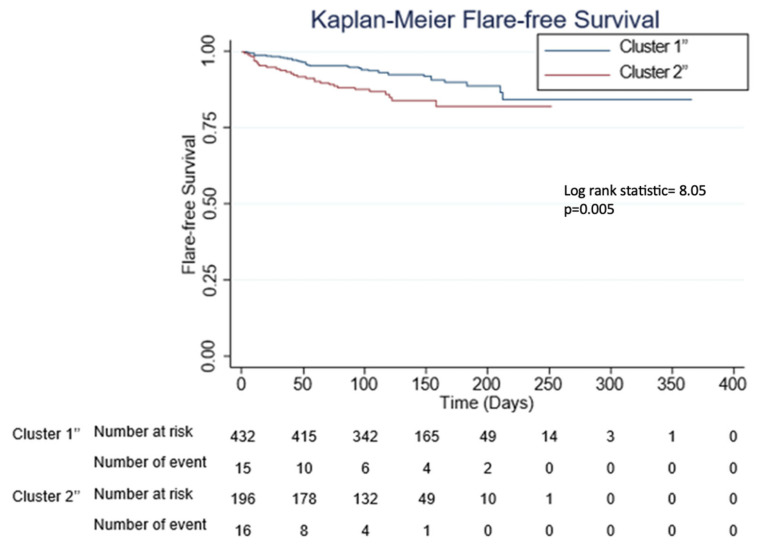
Kaplan–Meier curves for flare-free survival according to the different phenotypic clusters. C1″ and C2″ denote first and second clusters of the combined cohort, respectively.

Latent class analysis performed using the combined cohort of 633 patients recreated the SLE clusters (C1″ and C2″) with similar characteristics compared to the test and validation cohorts (Supplementary Table S1, Table 2 and Table 3). Of note, a higher proportion of patients in C2″ had immunomodulatory/immunosuppressive treatment interruption for vaccination as compared to patients in C1″ (6.6% vs. 0.2%) (*p* < 0.001). Similarly, C2″ had more haematological flares compared to C1″ (5.6% vs. 2.1%) (*p* = 0.020). On Cox regression analysis, C2″ had shorter time to flare after the first dose of COVID-19 mRNA vaccination as compared to C1″ with a hazard ratio (HR) of 1.4, 95% confidence interval (CI) 1.1–1.9, *p* = 0.014, after adjusting for flare after dose one vs. two (Table 4). A Kaplan–Meier curve depicting flare-free survival was plotted and is shown in Figure 3. 

### 3.5. Baseline Characteristics of the Major Ethnic Groups in the Combined Cohort

A comparison of the input variables used for the LCA modelling revealed that among the major ethnic groups in Singapore, Malays were predominantly represented in C2″ (*p* < 0.001) and they were younger (*p* < 0.001) and required higher doses of prednisolone (*p* = 0.001) (Table 5). There were also more males with Malay ethnicity, although not statistically significant (*p* = 0.200). SLE patients of other ethnicities were also overrepresented in C2″ (*p* < 0.001) and these patients were younger (*p* < 0.001). 

## 4. Discussion

For patients with autoimmune diseases including SLE, COVID-19 vaccination has been encouraged based on the increased risk and severe outcome of infection, and preliminary reports that mRNA COVID-19 vaccination was not associated with increased disease flares [21,22,23]. Identification of contributing factors for SLE flares after COVID-19 vaccination has been hampered by the relative dearth of large-scaled, adequately powered studies.

In this study, through the innovative approach of using LCA, we found two distinct phenotypic clusters of SLE with different patterns of flares following mRNA COVID-19 vaccination. The main finding of our study was that a cluster of younger, non-Chinese, male SLE patients with higher baseline disease activity tended to flare, particularly with haematological flares, after vaccination. Accordingly, more SLE patients in the second cluster were on background medications such as hydroxychloroquine, prednisolone > 7.5 mg/day, mycophenolate mofetil and tacrolimus. On Cox regression analysis, adjusting for flare after one or two doses of mRNA COVID-19 vaccination, C2″ had shorter time to flare as compared to C1″ (HR 1.4, 95% CI 1.1–1.9, *p* = 0.014). To our knowledge, this is the first study to employ LCA in SLE patients to identify different phenotypes predictive of SLE flares after mRNA COVID-19 vaccination. We have reported previously that younger age, non-Chinese ethnicity and male gender are associated with higher disease activity [24]. It is thus interesting to observe increased SLE flares following mRNA COVID-19 vaccination in patients with these characteristics likely due to higher baseline disease activity, further contributed to by treatment interruption for vaccination.

Various studies have reported associations between lupus disease flares and mRNA COVID-19 vaccination (Supplementary Table S3) [25,26,27,28,29] and two have focused on SLE patients [25,26]. A study involving 183 SLE patients reported that 11 (6%) patients experienced flares following vaccination, most of which were mild and constituted symptoms such as joint pain, skin rash, fatigue, muscle aches and mouth ulcers [25]. A multicentre cohort study of 452 SLE patients who had received COVID-19 vaccination found that 4% of patients flared after immunisation, with musculoskeletal, constitutional symptoms and renal being the most common types of flare; however, none of the disease flares required hospitalisation [26]. It is worth noting that new-onset SLE after mRNA COVID-19 vaccination has been described in various case reports, raising the plausibility of an association between induced autoimmunity and mRNA vaccination [30,31]. We recently reported that up to 9% of SLE patients experience disease flares following mRNA COVID-19 vaccination in a multi-ethnic Southeast Asian cohort [17]. Given the heterogeneity of SLE, identification of phenotypic clusters of patients at risk of flares after vaccination is an important question in need of through investigation. 

The difference in ethnic representation between the clusters is intriguing and suggests that non-Chinese ethnicity may be associated with unique characteristics to impact flares after mRNA COVID-19 vaccination. Southeast Asian countries such as Indonesia, Malaysia and Singapore have three major ethnic groups due to their immigration history. In 2020, Chinese, Malays and Indians constituted 74.3%, 13.5% and 9.0%, respectively, of the resident Singapore population [32]. In our earlier meta-analysis of Southeast Asian SLE patients, there were no significant differences in clinical manifestations between Malay and non-Malay ethnicities [24]. In the largest multiethnic comparison of SLE from Malaysia, Malay patients tended to be younger and of male gender [33], which is in keeping with our current findings (Table 5). In addition, in a recent cross-sectional survey of SLE patients from Singapore, we reported that Malays were more likely to be in the cluster to be worried about COVID-19 and modify their immunomodulatory/immunosuppressive treatment, findings that are also replicated here [34]. Higher doses of prednisolone in Malay SLE patients are also indicative of higher disease activity (Table 5). Hence, younger, male SLE patients of Malay ethnicity with higher disease activity may drive the clustering of our dataset and it will be important for physicians to identify this group of patients for closer monitoring immediately before and after mRNA COVID-19 vaccination. Lastly, ethnic Malay SLE patients in Singapore may also represent an ethnic minority with disadvantaged socioeconomic profiles, similar to other countries [35,36]. 

This study has several strengths. First, to the best of our knowledge, this is the largest study of post-mRNA COVID-19 vaccination flares in SLE patients to date. This is also the only Asian-focused study investigating the association of COVID-19 mRNA vaccination and risk of SLE flares. Second, we included SLE patients from eight of nine public hospitals in Singapore. As vaccination data were obtained centrally through the National Immunisation Register, this represents a near complete capture of all eligible SLE patients in Singapore. Third, we included a pre-determined test and validation cohort to identify phenotypic clusters of SLE patients. Although the test and validation cohorts were slightly different in terms baseline clinical characteristics, LCA identified similar phenotypic clusters of patients that predisposed to disease flares following mRNA COVID-19 vaccination. Further, LCA performed on the combined cohort recreated similar clusters, lending support to the robustness of our cluster analysis model. Using LCA, we identified two distinct phenotypic clusters of SLE with different patterns of flares following mRNA COVID-19 vaccination which would not have been discerned by any other multivariate statistical method [37]. Limitations of this study include the lack of long-term follow up data, so we are unable to determine the longitudinal stability of the clusters identified. This retrospective study is also unable to account for suboptimal medication adherence and dose changes by patients themselves, if not reported to the attending physician. In a similar vein, SLE flares were only captured if reported to and documented by the treating rheumatologist in the medical records. It is possible that some flares were not recorded by the rheumatologist. Another limitation is that the date of flare was largely approximated from the medical records. Lastly, the focus of this study being on a multi-ethnic Southeast Asian SLE cohort may limit generalizability of our findings to patients of other ancestries ethnicities, hence warranting validation in other lupus cohorts of different racial compositions.

Patients with SLE have exhibited hesitancy in uptake of COVID-19 vaccination, often due to safety concerns, especially the risk of flare of disease after vaccination [8,38]. To overcome such barriers and promote vaccine uptake, understanding the associations between disease flare and vaccination is warranted. As the world progresses towards co-existing with the SARS-CoV-2 pathogen, physicians caring for patients at high-risk such as SLE should be well informed regarding the risks and benefits of vaccination to counsel and manage patients accordingly. The role of routine vaccinations in SLE patients is well-established especially given that their immunocompromised states render them susceptible to developing severe sequaelae from infections [39,40,41]. For instance, routine vaccinations against influenza and streptococcus pneumoniae have been found to be generally safe, well-tolerated and efficacious in SLE patients [39]. Although initial clinical trials of COVID-19 vaccinations have largely excluded immunocompromised people such as those with SLE, real world data in this patient population have yielded reassuring results, having been found to contribute to strong and sustained humoral immune-responses [42,43], proving to be generally safe and efficacious in reducing the risk of COVID-19 [44,45,46,47,48], and improving outcomes including hospitalisation and mortality (Supplementary Table S3) [25,26,27,28,29].

## 5. Conclusions

While mRNA COVID-19 vaccination uptake in SLE patients has been hindered by concerns of suboptimal efficacy of vaccination when on immunosuppressive treatment and the possibility of disease flare post-vaccination [34], this study shows that vaccination remains generally safe. Physicians are recommended to dispel their fears and facilitate informed decision making. While mRNA COVID-19 vaccination should be strongly encouraged in SLE patients, caution has to be exercised in monitoring for post-vaccination flares in patients with risk factors for flares including those of ethnic minority, young age, male gender and suboptimal disease control at time of vaccination.

## Figures and Tables

**Table 1:** Baseline characteristics of SLE patients. Data are presented as n (%) or median (IQR).

	Combined Cohort (n = 633)	Test Cohort (n = 302)	Validation Cohort (n = 331)	
Variables	n (%) or Median (IQR)	n (%) or Median (IQR)	n (%) or Median (IQR)	*p*-Value
Race				0.424
Chinese	474 (74.9)	235 (77.8%)	239 (72.2%)	
Malay	90 (14.2)	38 (12.6%)	52 (15.7%)	
Indian	29 (4.6)	13 (4.3%)	16 (4.8%)	
Others	40 (6.3)	16 (5.3%)	24 (7.3%)	
Age (years)	52 (38–64)	46 (32–60)	58 (46–67)	<0.001
Sex				
Male	72 (11.4)	39 (12.9%)	33 (10.0%)	0.244
Vaccine type Pfizer/Biontech	575 (90.8)	282 (93.4%)	293 (88.5%)	0.034
Moderna	58 (9.2)			
Previous COVID-19 infection	10 (1.6)	3 (1.0%)	7 (2.1%)	0.345
Treatment interruption	14 (2.2)	0 (0.0%)	14 (4.2%)	<0.001
Disease activity				0.007
Remission	329 (52.0)	175 (57.9%)	154 (46.5%)	
Low	264 (41.7)	114 (37.7%)	150 (45.3%)	
Moderate	36 (5.7)	13 (4.3%)	23 (6.9%)	
High	4 (0.6)	0 (0.0%)	4 (1.2%)	
Baseline immunosuppression				
Prednisolone dose > 7.5 mg/day	51 (8.1)	17 (5.6%)	34 (10.3%)	0.032
Hydroxychloroquine	519 (82.0)	236 (78.1%)	283 (85.5%)	0.016
Mycophenolate mofetil	155 (24.5)	47 (15.6%)	108 (32.6%)	<0.001
Azathioprine	85 (13.4)	56 (18.5%)	29 (8.8%)	<0.001
Methotrexate	35 (5.5)	19 (6.3%)	16 (4.8%)	0.423
Tacrolimus	15 (2.4)	6 (2.0%)	9 (2.7%)	0.545
Cyclosporin	13 (2.1)	11 (3.6%)	2 (0.6%)	0.007
Sulfasalazine	9 (1.4)	3 (1.0%)	6 (1.8%)	0.509
Leflunomide	2 (0.3)	2 (0.7%)	0 (0.0%)	0.227
Rituximab	2 (0.3)	0 (0.0%)	2 (0.6%)	0.500
Cyclophosphamide	1 (0.2)	0 (0.0%)	1 (0.3%)	1.000

**Table 2:** Cluster baseline characteristics of test, validation and combined cohorts. Data are presented as n (%) or median (IQR).

	Test Cohort (n = 302)			Validation Cohort (n = 331)			Combined Cohort (n = 633)		
Cluster	1 n = 250	2 n = 52	*p* Value	1′ n = 183	2′ n = 148	*p* Value	1″ n = 435	2″ n = 198	*p* Value
Variables									
Race			<0.001			<0.001			<0.001
Chinese	204 (81.6)	31 (59.6)		153 (83.6)	86 (58.1)		363 (83.5)	111 (56.1)	
Malay	29 (11.6)	9 (17.3)		19 (10.4)	33 (22.3)		42 (9.7)	48 (24.2)	
Indian	7 (2.8)	6 (11.5)		5 (2.7)	11 (7.4)		16 (3.7)	13 (6.6)	
Others	10 (4.0)	6 (11.5)		6 (3.3)	18 (12.2)		14 (3.2)	26 (13.1)	
Age, median (IQR)	60	41.5	<0.001	58	31	<0.001	60	31	<0.001
	(49–69)	(31.5–53.5)		(49–66)	(22–38)		(51–67)	(24–38)	
Sex									0.043
Male	27 (10.8)	12 (23.1)	0.023	11 (6.0)	22 (14.9)	0.009	42 (9.7)	30 (15.2)	
Vax type Pfizer/Biontech	232 (92.8)	50 (96.2)	0.376	158 (86.3)	135 (91.2)	0.166	394 (90.6)	181 (91.4)	0.734
Previous COVID-19 infection	3 (1.2)	0 (0.0)	0.427	5 (2.7)	2 (1.4)	0.385	8 (1.8)	2 (1.1)	0.438
Treatment interruption	0 (0.0)	0 (0.0)	-	1 (0.6)	13 (8.8)	<0.001	1 (0.2)	13 (6.6)	<0.001
Disease activity			<0.001			<0.001			<0.001
Remission	174 (69.6)	1 (1.9)		90 (49.2)	64 (43.2)		244 (56.1)	85 (42.9)	
Low	72 (28.8)	42 (80.8)		89 (48.6)	61 (41.2)		175 (40.2)	89 (45.0)	
Moderate	4 (1.6)	9 (17.3)		4 (2.2)	19 (12.8)		16 (3.7)	20 (10.1)	
High	0 (0.0)	0 (0.0)		0 (0.0)	4 (2.7)		0 (0.0)	4 (2.0)	
Baseline immunosuppression									
Prednisolone dose > 7.5 mg/day	4 (1.6)	13 (25.0)	<0.001	2 (1.1)	32 (21.6)	<0.001	12 (2.8)	39 (19.7)	<0.001
Hydroxychloroquine	185 (74.0)	51 (98.1)	<0.001	149 (81.4)	134 (90.5)	0.027	336 (77.2)	183 (92.4)	<0.001
Mycophenolate mofetil	37 (14.8)	10 (19.2)	0.423	41 (22.4)	67 (19.2)	<0.001	77 (17.7)	78 (39.4)	<0.001
Azathioprine	47 (18.8)	9 (17.3)	0.801	14 (7.7)	15 (45.3)	0.427	59 (13.6)	26 (13.1)	0.883
Methotrexate	9 (3.6)	10 (19.2)	<0.001	12 (6.6)	4 (2.7)	0.104	28 (6.4)	7 (3.5)	0.139
Tacrolimus	0 (0.0)	6 (11.5)	<0.001	0 (0.0)	9 (6.1)	0.001	3 (0.7)	12 (6.1)	<0.001
Cyclosporin	1 (0.4)	10 (19.2)	<0.001	1 (0.6)	1 (0.7)	0.880	7 (1.6)	6 (3.0)	0.242
Sulfasalazine	1 (0.4)	2 (3.8)	0.078	2 (1.1)	4 (2.7)	0.275	3 (0.7)	6 (3.0)	0.030
Leflunomide	2 (0.8)	0 (0.0)	0.518	0 (0.0)	0 (0.0)	-	2 (0.5)	0 (0.0)	0.339
Rituximab	0 (0.0)	0 (0.0)	-	0 (0.0)	2 (1.4)	0.115	0 (0.0)	2 (1.1)	0.098
Cyclophosphamide	0 (0.0)	0 (0.0)	-	0 (0.0)	1 (0.7)	0.265	0 (0.0)	1 (0.5)	0.138

C1, C1′ and C1″ denote the first cluster of test, validation and combined cohorts, respectively. C2, C2′ and C2″ denote the second cluster of the test, validation and combined cohorts, respectively.

**Table 3:** Cluster flare characteristics of test, validation and combined cohorts. Data are presented as n (%) or median (IQR).

	Test Cohort (n = 302)			Validation Cohort (n = 331)			Combined Cohort (n = 633)		
Cluster	1 n = 250	2 n = 52	*p* Value	1′ n = 183	2′ n = 148	*p* Value	1″ n = 435	2″ n = 198	*p* Value
Variables									
Flare	19 (7.6)	9 (17.3)	0.036	17 (9.3)	24 (16.2)	0.066	39 (9.0)	30 (15.2)	0.021
Organ system									
General	3 (1.2)	2 (3.8)	0.206	1 (0.5)	3 (2.0)	0.328	5 (1.1)	4 (2.0)	0.471
Haematological	2 (0.8)	3 (5.8)	0.037	7 (3.8)	8 (5.4)	0.492	9 (2.1)	11 (5.6)	0.020
Musculoskeletal	11 (4.4)	5 (9.6)	0165	4 (2.2)	8 (5.4)	0.119	17 (3.9)	11 (5.6)	0.350
Mucocutaneous	3 (1.2)	1 (1.9)	0.532	3 (1.6)	6 (4.1)	0.308	7 (1.6)	6 (3.0)	0.242
Cardiorespiratory	1 (0.4)	1 (1.9)	0.315	1 (0.5)	1 (0.7)	1.000	2 (0.5)	2 (1.0)	0.593
Renal	1 (0.4)	1 (1.9)	0.315	7 (3.8)	5 (3.4)	0.829	8 (1.8)	6 (3.0)	0.385
Gastrointestinal	0 (0.0)	0 (0.0)	-	0 (0.0)	1 (0.7)	0.447	0 (0.0)	1 (0.5)	0.313
Vasculitis	2 (0.8)	0 (0.0)	1.000	1 (0.5)	1 (0.7)	1.000	4 (0.9)	0 (0.0)	0.315
Others	1 (0.4)	0 (0.0)	1.000	1 (0.5)	0 (0.0)	1.000	2 (0.5)	0 (0.0)	1.000
Flare severity			0.034			0.020			0.022
No flare	231 (92.4)	43 (82.7)		165 (90.7)	124 (83.8)		395 (90.8)	168 (84.5)	
Mild/self-limiting	7 (2.8)	1 (1.9)		2 (1.1)	3 (2.0)		10 (2.3)	3 (1.5)	
Mild/moderate	10 (4.0)	7 (13.5)		14 (7.7)	12 (8.1)		24 (5.5)	19 (9.6)	
Severe	2 (0.8)	1 (1.9)		1 (0.5%)	9 (6.1)		5 (1.2)	8 (4.0)	
Time to flare			0.065			0.122			0.012
Within 90 days after first dose	10 (4.0)	6 (11.8)		11 (6.1)	18 (12.2)		22 (5.1)	23 (11.7)	
Beyond 90 days	9 (3.6)	2 (3.9)		5 (2.8)	5 (3.4)		15 (3.5)	6 (3.0)	
No flare	231 (92.4)	43 (84.3)		165 (91.2)	124 (84.4)		395 (91.4)	168 (85.3)	
Median time to flare	59.0 (34.0–154.0)	55.5 (21.0–96.8)	0.449	52.5 (32.5–111.0)	40.0 (12.0–75.0)	0.217	53.0 (35.5–115.0)	42.0 (12.5–76.5)	0.072
Incidence of flare per 1000 patient-month, (95% CI)	5.4 (3.3–8.5)	12.4 (5.7–23.5)	0.036	6.7 (3.9–10.7)	11.6 (7.4–17.2)	0.066	6.4 (4.6–8.8)	10.8 (7.3–15.4)	0.021
Hospital admission	2 (0.8)	1 (1.9)	0.434	2 (1.1)	7 (4.7)	0.084	5 (1.1)	7 (3.5)	0.057
Improved disease activity	35 (14.0)	10 (19.2)	0.335	16 (8.7)	11 (7.4)	0.692	54 (12.4)	18 (9.1)	0.222

C1, C1′ and C1″ denote the first cluster of test, validation and combined cohorts, respectively. C2, C2′ and C2″ denote the second cluster of the test, validation and combined cohorts, respectively.

**Table 4:** Cox regression for time to flare after mRNA COVID-19 vaccination in the combined cohort.

Variables	Unadjusted		Adjusted *	
	HR (95% CI)	*p*-Value	HR (95% CI)	*p*-Value
Cluster				
1″	1.0		1.0	
2″	2.0 (1.9–2.1)	<0.001	1.4 (1.1–1.9)	0.014

C1″ and C2″ denote first and second clusters of the combined cohort, respectively. * Adjusted for flare after dose 1 or 2. * In the adjusted model, 96.8% of subjects (613 out of 633 subjects) were used.

**Table 5:** Cluster distribution and baseline characteristics among the different ethnic groups. Data are presented as n (%) or median (IQR).

	Chinese n = 474 (74.9%)	Malay n = 90 (14.2%)	Indian n = 29 (4.6%)	Others n = 40 (6.3%)	*p* Value
Cluster distribution					<0.001
Cluster 1″	363 (76.6)	42 (46.7)	16 (55.2)	14 (35.0)	
Cluster 2″	111 (23.4)	48 (53.3)	13 (44.8)	26 (65.0)	
Age, median (IQR)	55.0 (42.0–65.3)	42.0 (27.8–62.3)	49.0 (35.5–61.0)	41.5 (32.3–50.1)	<0.001
Sex Male	56 (11.8)	13 (14.4)	2 (6.9)	1 (2.5)	0.200
Vax type Pfizer/Biontech	433 (91.4)	82 (91.1)	26 (89.7)	34 (85.0)	0.534
Previous COVID-19 infection	7 (1.5)	2 (2.2)	1 (3.4)	0 (0.0)	0.458
Treatment interruption	8 (1.7)	5 (5.6)	0 (0.0)	1 (2.5)	0.123
Disease activity					0.383
Remission	252 (53.2)	43 (47.8)	12 (41.4)	22 (55.0)	
Low	194 (40.9)	41 (45.6)	15 (51.7)	14 (35.0)	
Moderate	26 (5.5)	5 (5.6)	1 (3.4)	4 (10.0)	
High	2 (0.4)	1 (1.1)	1 (3.4)	0 (0.0)	
Baseline immunosuppression					
Prednisolone dose > 7.5 mg/day	27 (5.7)	16 (17.8)	4 (13.8)	4 (10.0)	0.001
Hydroxychloroquine	388 (81.9)	73 (81.1)	25 (86.2)	33 (82.5)	0.938
Mycophenolate mofetil	116 (24.5)	25 (27.8)	3 (10.3)	11 (27.5)	0.277
Azathioprine	65 (13.7)	16 (17.8)	0 (0.0)	4 (10.0)	0.060
Methotrexate	25 (5.3)	4 (4.4)	4 (13.8)	2 (5.0)	0.278
Tacrolimus	8 (1.7)	3 (3.3)	0 (0.0)	4 (10.0)	0.021
Cyclosporin	10 (2.1)	2 (2.2)	1 (3.4)	0 (0.0)	0.713
Sulfasalazine	5 (1.1)	1 (1.1)	2 (6.9)	1 (2.5)	0.076
Leflunomide	2 (0.4)	0 (0.0)	0 (0.0)	0 (0.0)	1.000
Rituximab	1 (0.2)	1 (1.1)	0 (0.0)	0 (0.0)	0.440
Cyclophosphamide	0 (0.0)	1 (1.1)	0 (0.0)	0 (0.0)	0.251

C1″ and C2″ denote first and second clusters of the combined cohort, respectively.

## Data Availability

The authors confirm that the data supporting the findings of this study are available within the article and its Supplementary Materials.

## Supplementary Materials

The following supporting information can be downloaded at: https://www.mdpi.com/article/10.3390/vaccines12010029/s1, Table S1: Latent Class Analysis model fit (Bayesian information criterion) for test, validation and combined clusters; Table S2: Proportion of patients who flared within 3 weeks of the first dose of mRNA COVID-19 vaccination versus within 3 weeks of the second dose; Table S3: Studies on SLE flares after mRNA COVID-19 vaccination.
