# Supplementary material for: Latent Class Analysis Identifies Distinct Phenotypes of Systemic Lupus Erythematosus Predictive of Flares after mRNA COVID-19 Vaccination: Results from the Coronavirus National Vaccine Registry for ImmuNe Diseases SINGapore (CONVIN-SING)
Source: Vaccines (Basel). 2023 Dec 27;12(1):29. doi: 10.3390/vaccines12010029 (PMC10819486; doi:10.3390/vaccines12010029)
Supplement: Supplementary file 1 [file vaccines-12-00029-s001.zip › vaccines-2697247-supplementary.pdf]

SUPPLEMENTARY INFORMATION

Latent Class Analysis Identifies Distinct Phenotypes of Systemic Lupus Erythematosus Predictive of Flares After mRNA COVID-19 Vaccination: Results from the COronavirus National Vaccine registry for ImmuNe diseases SINGapore (CONVIN-SING)

Tao Ming Sim, Manjari Lahiri, Margaret Ma, Peter Pak-Moon Cheung, Anselm Mak, Warren Fong, Stanley Angkodjojo, Chuanhui Xu, Kok Ooi Kong, Thaschawee Arkachaisri, Kee Fong Phang, Teck Choon Tan, Qai Ven Yap, Yiong Huak Chan, Melonie Sriranganathan, Tyng Yu Chuah, Nur Emillia Roslan, Yih Jia Poh, Annie Law, Amelia Santosa and Sen Hee Tay

Supplementary Table S1. Latent Class Analysis model fit (Bayesian information criterion) for test, validation and combined clusters.

Class	Test Cohort	Validation Cohort	Combined Cohort
2	5348.407	6230.895	11577.99
3	5396.743	6274.162	11649.08
4	5396.517	6267.334	11624.43
5	5455.038	6372.67	11728.93

Supplementary Table S2. Proportion of patients who flared within 3 weeks of the first dose of mRNA COVID-19 vaccination versus within 3 weeks of the second dose.

Cohort	Flare after dose 1	Flare after dose 2	P value
Test	5/301 (1.7%)	4/296 (1.4%)	0.756
Validation	11/328 (3.4%)	11/317 (3.5%)	0.935
Combined	16/629 (2.5%)	15/613 (2.4%)	0.913

Supplementary Table S3. Studies on SLE flares after mRNA COVID-19 vaccination.

Patients	Number	Ethnicity	Design	COVID-19 mRNA Vaccine	Results	Notes	Ref
Outpatient SLE from a rheumatology division in New York	SLE: 183	White: 65.6%<br><br>Hispanic: 15.9%<br><br>Others: 18.5%	Cross-sectional study	Pfizer and Moderna	11 (6%) patients reported flares, mostly mild-moderate in severity	Common manifestations of flare include joint pain, skin rash, fatigue, muscle aches and mouth ulcers	25
SLE patients who had received COVID-19 vaccination	SLE: 452	Italian majority	Retrospective cohort study	Pfizer and Moderna	19 (4%) patients experienced flares, none required hospitalization	Musculoskeletal, constitutional symptoms and renal were the most common type of flare	26
Patients with rheumatic and musculoskeletal diseases who had received two doses of COVID-19 mRNA vaccination	SLE: 273<br><br>Total: 1,377	White: 90%<br><br>Non-white: 10%	Prospective cohort study	Pfizer and Moderna	30 (11%) SLE patients experienced flares, none required hospital or intensive care unit admission	Common manifestations of flare include joint pain, fatigue, keratoconjunctivitis sicca and musculoskeletal pain	27
Patients with systemic rheumatic diseases who had received COVID-19 vaccination	SLE: 391<br><br>Total: 2,860	Overall cohort:<br><br>White: 86.3%<br><br>Non-white: 13.7%	Cross-sectional study	Pfizer and Moderna	Flare of any rheumatic disease was as high as 13.1%, but significant flares requiring changes to medication were relatively uncommon (4.2%)	While rates of flares of rheumatic disease and systemic reactions to mRNA COVID-19 vaccination were reported, flares severe enough to warrant a change in medication were uncommon	28
Patients with inflammatory and non-inflammatory rheumatic and musculoskeletal disease who had received COVID-19 vaccination	SLE: 367<br><br>Total: 5,121	White majority, multinational cohort from various European countries	Retrospective cohort study	Pfizer and Moderna	About 5% of patients with inflammatory rheumatic musculoskeletal disease flared following vaccination, 1.7% were mild/minor, 2.6% were moderate and 0.5% were severe	Common manifestations of flare include polyarthralgia, arthritis flare and fatigue<br><br>Overall, mRNA COVID-19 vaccination was largely well-tolerated	29

					37% of patients experienced vaccine-related adverse effects, mostly non-severe		
